# A Baldwin interpretation of adult hippocampal neurogenesis: from functional relevance to physiopathology

**DOI:** 10.1038/s41380-021-01172-4

**Published:** 2021-06-08

**Authors:** Djoher Nora Abrous, Muriel Koehl, Maël Lemoine

**Affiliations:** 1 Univ. Bordeaux, INSERM, Neurocentre Magendie, U1215, Neurogenesis and Pathophysiology group, F-33000 Bordeaux, France; 2 University Bordeaux, CNRS, ImmunoConcEpT, UMR 5164, Bordeaux, France

**Keywords:** Neuroscience, Psychiatric disorders

## Abstract

Hippocampal adult neurogenesis has been associated to many cognitive, emotional, and behavioral functions and dysfunctions, and its status as a selected effect or an “appendix of the brain” has been debated. In this review, we propose to understand hippocampal neurogenesis as the process underlying the “Baldwin effect”, a particular situation in evolution where fitness does not rely on the natural selection of genetic traits, but on “ontogenetic adaptation” to a changing environment. This supports the view that a strong distinction between developmental and adult hippocampal neurogenesis is made. We propose that their functions are the constitution and the lifelong adaptation, respectively, of a basic repertoire of cognitive and emotional behaviors. This lifelong adaptation occurs through new forms of binding, i.e., association or dissociation of more basic elements. This distinction further suggests that a difference is made between developmental vulnerability (or resilience), stemming from dysfunctional (or highly functional) developmental hippocampal neurogenesis, and adult vulnerability (or resilience), stemming from dysfunctional (or highly functional) adult hippocampal neurogenesis. According to this hypothesis, developmental and adult vulnerability are distinct risk factors for various mental disorders in adults. This framework suggests new avenues for research on hippocampal neurogenesis and its implication in mental disorders.

## Functional relevance of adult hippocampal neurogenesis

### The evolutionary function of adult hippocampal neurogenesis

The existence, nature, and function of adult neurogenesis (AN) in some discrete areas of the central nervous system has been controversial almost since its discovery by Altman and Das [1, 2]. Altman did not investigate the function of these neurons, called “microneurons” due to their small size, but proposed that they could be “the modulatory and plastic elements” of the animal’s response to its “varied external environment” [2]. Some on the opposite side have suggested that AN has no function at all and is only a vestigial trait of tissue repair––“the appendix of the brain”, which would be useless, or almost useless [3], in animals with complex brains after neurodevelopment is achieved [4–6].

In this review, we propose to clarify the status of AN as a function and to draw some important consequences for its study. First, two theoretical arguments in this debate must be clarified. The first one revolves around the definition of the term “function”. From the evolutionary perspective, the question of whether AN has a function depends in part on whether natural selection has been fashioning it. Because levels of AN in mammals are much lower than levels in less complex species, it seems clearly vestigial [7, 8], and it is thus hard to claim that it has a function in the sense of an evolutionary selected effect. If then, AN is “atavistic”, or “vestigial”, it can only have a physiological function, i.e. it just happens to play a useful causal role. Supporting this view, many experiments annihilating AN result in various serious impairments [9], which suggests that AN has a function in the sense of a causal physiological role. Some, however, would be happy to say that it is only an instance of exaptation, that is, a beneficial side effect that was not selected for [10]. The second argument revolves about individualization. Indeed, AN is often proposed to play a role in individualization through various forms of flexibility in the face of enriched environment, as recently emphasized by Kempermann [11]. Individualization involves the acquisition of non-hereditary traits, and a trait with no transmissible effects cannot be naturally selected. Some individuals could use this flexibility to their advantage without it being a function in the evolutionary sense, which again leads to hesitations as to whether AN has a function.

In this review, we defend the view that AN is indeed a selected effect and has a function in the evolutionary sense of the word. However, this function must be understood in a particular context, that of the so-called “Baldwin effect”, i.e., a situation in evolution where a particular function will not disappear, but cannot be genetically hardwired either, so that it has to rely on individual adaptation, repeated over and over again with each generation. To understand this point of view, it must first be noted that AN is likely to have been pleiotropic at some point in evolution. Its first function has very likely been repair of tissue in simple brains where neurons with relatively few connections are more easily replaceable [12]. On the contrary, the more complex a brain is, the less easily repairable it becomes. Thus, there may have been a point in evolution when the advantages of complexity in certain environments have overcome the advantages of reparability. At this point, AN must have lost its tissue repair function. This does not involve that it has lost any function. It may indeed have regressed in almost all parts of the brain, except in certain privileged areas, where its maintenance at a certain low level increased the fitness of individuals in spite of the costs associated to keeping neurogenesis. This is the case in the dentate gyrus (DG) of the hippocampus.

What, then, can the function of adult hippocampal neurogenesis (AHN) be? Environments contain infinitely diverse and changing cues that should or should not trigger behaviors. These cues can be learnt and updated by the same individual, but any genetically programmed behavioral response would probably not be adapted to most cases. The Baldwin effect, as we interpret it, is the specific case of such an evolutionary function, stuck in a genetic dead-end of evolution. This function cannot progress into genetic hardwiring, but it will not regress either. At the level of species, the pressure towards the disappearance of AHN would in fact come from the competition with hardwired genetic variations that would automatize the adapted behavior whenever possible, while the pressure towards its maintenance would come from a certain level of change in the environment. This would also explain the various levels of AHN found in different wild species, mammals in particular [13], but not exclusively [12]. In short, the capacity to learn can evolve, but not the learnt behaviors themselves. At the level of the individual, the Baldwin effect sets the stage for what Baldwin called “ontogenetic adaptation”, that is, a demand on individuals to adopt adaptive behaviors they are not genetically equipped to produce automatically. In adults, the ability to adapt behaviors relies, we propose, on AHN.

This interpretation has important consequences with regard to mental disorders. Instead of being exceptional anomalies, they could be as endemic in some species and environments as bad digestion is in pandas. Indeed, we suggest that the Baldwin effect produces a niche characterized by a high level of demand that makes failure very likely [14]. An important additional property of the enriched environment analyzed by Kempermann [11], is that only individual ontogenetic adaptation, not phylogenic genetic adaptation, can meet this level of demand. Such a niche, experimentally mimicked by most stress protocols, is very favorable to the development of mental disorders in the adult. Indeed, strong consistent data suggest that deficits in AHN are involved in most mental disorders that do not originate solely in neurodevelopmental dysfunctions: it is involved in anxiety disorders, post-traumatic stress disorder [15], addictions [16–18], and in some dysfunctions in the aging brain [19–21], but not in developmental disorders such as ADHD and autism spectrum disorder, or in schizophrenia spectrum and psychotic disorders.

Box 1 A potted history of the controversies around the Baldwin effect

- Baldwin (1861–1934) proposed that a mechanism of “ontogenetic adaptation” transmits acquired behaviors to the next generation, essentially through imitation, thus durably changing the social environment for next generations until the effects of learning are possibly replaced by hardwired, genetic determinants of similar behaviors [227].
- His theory has long been rejected as an attempt to re-introduce Lamarckism. In 1953, evolutionary biologist G. G. Simpson thus raised a paradox against the Baldwin effect: not only a mechanism of transmission through imitation would probably lower the selective pressure for the genetic equivalent to emerge rather than raise it, but the replacement by a rigid genetic mechanism would undermine the very goal of ontogenetic adaptation, namely, to show some behavioral flexibility [228].
- Defenders of the Baldwin effect emphasized its relevance to understand the rapid emergence of higher cognitive capacities, language in particular [229–231].
- A recent criticism of the Baldwin effect [232] is that it becomes useless in the broader theoretical framework of niche construction [233] recently proposed by evolutionary biologists.

### The physiological function of adult hippocampal neurogenesis

Evolutionary considerations are necessary but not sufficient to characterize the function of AHN. The hippocampus has been involved in diverse functions such as learning and memory, regulation of emotion, attentional processes and motivational states to name a few. It is a key structure for processing information about events and elements of context, the so-called episodic memory, and it is very sensitive to stress, drugs, and aging. The Baldwin hypothesis suggests that a clear distinction should be made between the genetically inherited behaviors that are eligible to adaptation, and the capacity to adapt them to current, changing circumstances. In line with it, we propose that developmental hippocampal neurogenesis (DHN), i.e., the formation of DG during development, underlies the initial setting up of a repertoire of basic, adaptable behaviors while AHN, i.e., the addition of new neurons in the DG during adulthood, underlies adult ontogenetic adaptation.

**Fig. 1:**
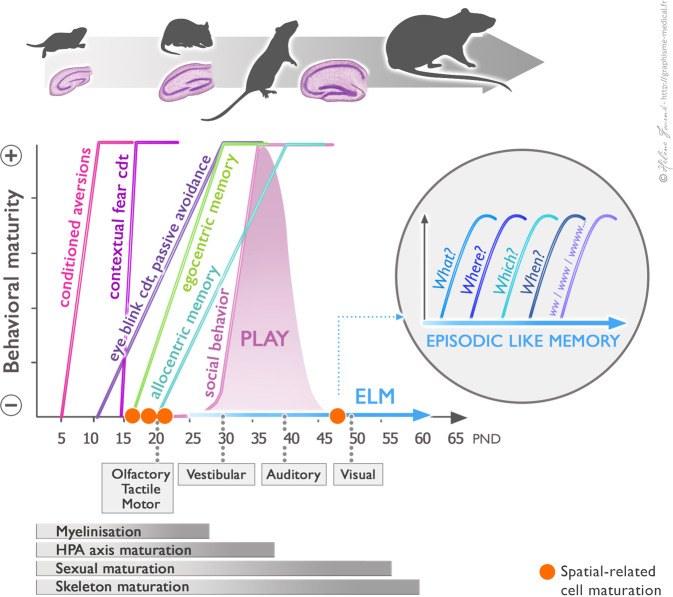
Schematic representation of maturation of behavioral capacities in relation to developmental milestones in rodents. The different ontogenetic stages illustrated are: the early postnatal period (first 3 weeks of life) divided into the neonatal period (first 2 weeks), the juvenile period (2nd and 3rd week postnatal), and the adolescent period (4th–8th week postnatal). Learning capacity emerges sequentially from the simple to the complex. This succession of distinct competencies are adaptive at a particular period characterized by the state of maturity of sensorimotor functions, hormonal system, and of cerebral structures. The hippocampal formation, and the DG in particular, a key structure involved in episodic memory, presents a protracted development. The red square represents the end of maturation of Boundary cells (PND16), Head direction cells (PND 19), Grid cells (PND 20) and Place cells (P50) in the hippocampal formation. Adapted from [24, 31, 32, 218–226].

In this context it is important to note that the development of memory and emotional responses are not singular ontogenetic processes [22–24] but that they follow a precise calendar (Fig. 1). Thus learning capacity emerges sequentially from the simple to the complex [25, 26] as illustrated in the following examples: (i) non-associative learning emerges before associative learning [27], (ii) simple conditioning (delay conditioning) emerges earlier than “high-order” (trace) conditioning [28, 29], (iii) the emergence of conditioned fear responses [30] precedes that of the conditioned eyeblink reflex [31], (iv) egocentric learning precedes allocentric learning in spatial navigation tasks [32], in which proximal cues are used before distal ones and reversal learning is the latest to emerge [33], and (v) novel objects (what) are recognized before locations (where), contexts (which), or their combination (what–where, what–which, what–where–which….) [32, 34, 35]. Interestingly, during the infant period, memories appear to be easily and rapidly forgotten, a process called “infantile amnesia” [22, 36]. Recent investigations have shown that infantile memories are in fact not lost and that the transition to adult-like memory (around postnatal day 25, PND25) is linked to the maturation of the hippocampus [37–41].

Although much less is known on the ontogeny of the emotional brain, the sequential addition of capacities has also been reported. Thus as early as the neonatal stage, rodents are able to manifest their threat/fear with ultrasonic vocalizations (USV with a peak at PND6–8) [42], and freezing behavior and unconditioned startle responses emerge later on during the second postnatal week [42]. Then, the typical development of fear follows a linear pattern with adolescence characterized by a period of heightened emotional reactivity [43–45]. The development of anxiety-like responses (i.e., behavioral responses occurring when an individual copes with a potentially dangerous situation and not with explicit immediate threats) seems to be delayed and emerges around weaning (PND21) to reach adult-like levels in young adult rats (PND56 [46]). Finally, behavioral inhibition for appetitive cues (sugar, drugs, novelty) or the ability to engage cognitive control in a sustained and controlled fashion maturate after adolescence [47, 48]. This period is also characterized by the maturation of social interaction with a peak in play behavior, increased impulsivity, and continued refinement of higher-order cognition [49].

Data on the functional relevance of DHN remaining very scarce, we speculate that these first-order capacities are associated with older neurons in the DG, the ones born during embryogenesis and the early postnatal period [50–52]. These capacities are probably submitted to selection, in the sense that individuals who do not acquire them are unlikely to survive long. However, there are also effects of low or disturbed DHN associated for example with altered developmental context (stress, etc.) that are likely to be seen only much later in the adult [53], with a probably limited effect on fitness.

During adulthood, new neurons are continuously added in the DG, and we propose that their function is to modulate and adapt the basic behaviors acquired during development, in the face of life events. With regards to hippocampal functioning, this is evidenced by the existence of binding capabilities allowing adult animals not only to recognize a specific object or a specific location but to be able to associate a specific item to an emotional valence and to a specific location, context and time (what where which when memory, i.e., an experienced-based memory). This involves the capacity to encode different sensory (visual, acoustic, olfactive) and interoceptive information, to link them in a unique representation, and to integrate them adequately (by comparing them to previously acquired ones) to support inferential reasoning [54, 55], i.e., the process by which existing memories are retrieved and recombined to cope with novel situations. It requires the flexible use of learned information and depends both on the ability to form distinct memory representations that share overlapping elements during encoding and on the ability to retrieve them from partial inputs.

The functions of DHN and of AHN are thus both different and interrelated. In the Baldwin hypothesis, the different behaviors associated to the formation of the hippocampus have one thing in common: they are the basic bricks of adult ontogenetic adaptation. We call this set of functions F1. According to our hypothesis, they should be DHN-associated, and broadly shared among individuals in a species. However, these basic behaviors make an individual adaptable, not necessarily adapted to its environment. For instance, the ability to trigger and extinct fear is necessary to adaptation, but not sufficient, as its occurrence in given circumstances may be maladapted. A second set of functions F2 is therefore related to the adaptation of these behaviors to circumstances, based on passed life events. These functions are AHN-associated. They are more specific to individuals, given the unique series of situations they have been through and what they have inferred from them. The rest of this section explores F2 into more detail.

#### AHN and memory processes

Episodic memory relies on the ability to form relational structures that bind spatial and non-spatial information to the specific spatial/temporal/emotional context in which an event takes place. During encoding, episodic memory relies on the ability to form distinct memory representations that share overlapping elements, a process called pattern separation (PS). We proposed in 2008 that the behavioral deficits exhibited by mice genetically depleted for AHN using multiple start locations in the watermaze might be interpreted as resulting from an impairment in spatial pattern separation [56]. Indeed, in this classical version of the watermaze that tests reference memory animals are required to link different spatial representations associated to the variable point of view at the departure. Spatial interferences may arise between juxtaposed or overlapped spatial memory representations. Thus, an increase in the amount of such interferences associated to an inability to extract and separate efficiently spatial representations may account for behavioral deficits during navigation in this task (for discussion see also [57]). A specific role for AHN in this process was demonstrated using a delayed nonmatching to place (DNMP) protocol that specifically challenges this function in a radial arm maze. In this task, animals had to select, from a choice of two arms, the arm location that had not been presented in a previous sample phase. The rewarded arm during the test phase varied in distance from the sample arm by a spatial separation of two, three, or four arms. When the distance was high the level of PS was low, and when the distance was low the level of PS was high. Ablating adult-born dentate granule neurons (Adu-DGNs) either by irradiation or by altering the neurogenic niche impaired the ability of the animals to discriminate the correct arm when its location was near the original location. Mice were also trained in a touchscreen test that measures the ability to choose the correct spatial location between two illuminated boxes that are separated far apart or are close [58]. A role of AHN in PS has been also proposed using the contextual fear conditioning task in which animals have to associate a context to an electric footshock. Mice were exposed to two similar contexts that shared features (A and B) and received footshocks in only one of them (A). Across the subsequent consecutive days mice were placed in counterbalanced order in the A-context and B-context conditioning chambers. In control mice training over several days enhanced discriminative performances, i.e., the association of the shock to the context (A). When AHN was genetically decreased mice did not improve their performance, and even lost their capability to discriminate the two contexts [59]. When AHN was genetically increased on the other hand, discriminative abilities were improved [60, 61]. From these data and others, PS has been assumed to be the core function of AHN [62]. However, the ability of mice to discriminate (or not) similar contexts or locations in close proximity does not necessarily reflect a PS process (for critical review see [63]). In addition, the deficits observed might result from a deficit of retrieving instead of encoding overlapping memories, a process called pattern completion [55, 64], and interestingly, the abilities to retrieve memory from partial cues/information is supposed to rely on relational coding. More importantly, this PS theory does not capture the contribution of AHN to all the functions that depend on it (for example drug-related memories), and the role of AHN most probably extends beyond the process of PS.

In accordance with the role of the DG in inferential reasoning, i.e., the process by which existing memories are retrieved and recombined to cope with novel situations (see above), we propose as an alternative hypothesis that Adu-DGNs establish relationships between multiple contextual elements that are not experienced together, and bind these relationships together in order to support inferential reasoning. One example to support this proposition: mice with ablated neurogenesis are deficient in learning the platform position in the classical version of the watermaze (reference memory) which requires to link different spatial representations associated to the variable point of view at the departure; this deficiency is certainly sustained by mice inability to link together the multiples scenes (associated with different departure points) in order to construct a cognitive map. In contrast their ability to find a hidden platform from a constant departure point is spared indicating that they are capable of place learning when they do not have to establish relationships between the different cues of the room. When they are requested to find the hidden platform (which position they have learned using constant departure training) starting from a novel location they are unable to use the learned information in a novel condition [56]. We thus propose that the binding capacities of Adu-DGNs are essential for flexible inferential memory expression when animals have to change strategy. These linking properties are also required for another type of inferential reasoning, i.e., reversal learning. In this case the platform position (and not the departure point) is changed and animals have to adapt their behavior to the novel experimental conditions [65].

In order to optimize the capacity for learning and memory, stored information are continuously degraded or cleared to avoid memory saturation and interferences from old memories (proactive interference). This process called “active forgetting” depends upon the production of new neurons after learning [66]. Interestingly, post-training increase of AHN only impacted recently acquired, and not remotely acquired, memories [67] suggesting that the addition of new neurons may alter the binding process. This destabilization could result from the competition of new added neurons with preexisting DGNs for their survival/connections [68, 69]. In addition, the destabilization of memories after post-training-increase of AHN (by running) facilitates the encoding of new, conflicting information (reversal under high level of interference) which indicates a reduction of proactive interference [70].

Another important aspect of episodic memory is its ability to perform mental time travel to execute episodic future thinking and to establish prospective memories. The very scarce evidence supporting a role of AHN in “time” processing is related to tasks that require integration of events that occur close in time [71–73] or to the ability of AHN to separate two events according to the amount of time elapsed between the two presentations [74, 75]. However, the involvement of AHN in memory for sequences of events that has been shown to depend upon the DG [76], and in prospective memory, awaits confirmation.

#### AHN and emotional states

The binding properties of AHN also explain previous behavioral studies ascribing a role of AHN in emotional states, whether positive or negative. When positive, emotions motivate to reach a goal or a reward. The DG supports reward process as shown by the existence of reward selective firing cells that coordinate CA3 neuronal activity to guide behavior [77]. Like natural reinforcers (food, physical and sexual activity), addictive drugs are highly rewarding and strengthen stimulus-response associations. Their reinforcing properties are usually assessed using the conditioning place preference (CPP) task. It consists of two equally-sized compartments that are clearly different from each other. During the conditioning phase, a compartment is associated to the drug (injected to the animals) and during the drug free test phase, preference for the drug-paired compartment is measured. Although ablating AHN does not impair the acquisition of drug‐induced conditioning, it increases the length of memories [18, 78] suggesting that the ability to associate the “reward” with the environment in which it was presented is increased. Because all addictive drugs (morphine, heroin, nicotine, methamphetamine, alcohol, cocaine) were reported to decrease AHN [79] we propose that low levels of Adu-DGNs, either linked to drug taking or achieved by experimental means, leads to an excessive binding by amplifying the weight of positive association between the cues and the drug that renders it less sensitive to the passage of time.

When negative, emotions have evolved as defenses and allow adapting to potential threats, as is the case of anxiety. According to Barlow’s definition, “anxiety” is defined as a future-oriented mood state in which one is not ready or prepared to cope with upcoming negative events. It consists in a complex cognitive, affective, physiological, and behavioral response system associated with preparation for the anticipated events or circumstances perceived as threatening [80]. Anxiety-related behaviors are classically studied either with approach-avoidance paradigms or defensive behaviors. The approach-avoidance paradigm exploits scenarios in which the environment (open and brightly lit) increases the risk for predation. Ablating AHN [81], decreasing Adu-DGNs survival [82] or silencing new neurons [83] has been shown to increase avoidance responses, indicating an increase in anxiety-like responses. Defensive reaction to the visual presence of a predator (rat) that was physically separated from the mice by a wire meshed wall was also increased after neurogenesis ablation [81]. Interestingly, safety signal learning (conditioned inhibition of fear) promotes the survival of Adu-DGNs that exerts an anxiolytic effect (decrease avoidance responses in a plus maze) [84]. Reciprocally, ablation of Adu-DGNs retards safety learning indicating that they provide information about the degree of threat or safety in the environment [84]. Altogether, these data suggest that AHN is required to efficiently integrate and bind the different sensory information coming from a potentially dangerous environment, and that its removal amplifies the weight of negative associations leading to an overreaction to aversive cues that are interpreted as threatening.

#### Functional specificity: DHN vs AHN

Contrasting to these complex functions, it is clear that Adu-DGNs do not sustain simple forms of spatial knowledge such as novelty detection [56, 85] or motivation for natural rewards [86–88] and that their ablation or inactivation does not resume the behavioral syndrome observed after lesion of the DG. For instance adult lesion induces locomotor hyperactivity [89], blocks the acquisition and expression of context-conditioned fear [90], and abolishes cocaine-induced CPP [91], which are not observed after manipulating AHN. In addition when animals with colchicine lesion of the DG have to learn to press for food contingent upon appropriate illumination of cue lamps (the positions of which provided a spatial component to the task), they present difficulty in directing motor activity specifically toward the S+ condition (bar pressing for food during the presentation of a green light) and withholding it during S− (red light) [89]. This contrasts with what is observed after AHN ablation that either does not impact [58, 92], or decrease [93] appetitive learning for food.

It could be argued that the discrepancies between DG lesions and AHN ablation result from differences in the size of the lesion, i.e., the number of DGNs that are removed. Although we cannot exclude this possibility, there are reasons to believe that they actually rely on the different functional, morphological, cellular and plastic properties of developmentally-born and adult-born neurons. Indeed, we and others have shown that manipulating the activity of a given DGNs population can produce or not behavioral deficits depending on the cell age but not on the number of tagged cells [52, 94] Furthermore, from a functional point of view, evidence points to important differences between developmentally and adult-born neurons. Thus, juvenile neurons have been shown to be important for the maturation of female–female social behavior as ablating DGNs from PND27 to PND35 decreased social exploration and time spent in close proximity with a conspecific, increased escape behavior, and impaired pup retrieval, all effects that were not seen after ablating Adu-DGNs [50]. As AHN has been involved in the maintenance of social memory [95–97], Juv-DGNs and Adu-DGNs neurons may actually play complementary roles in social behavior. In addition, we have shown that in contrast to Adu-DGNs, Neo-DGNs (PND7) are not recruited during spatial learning in the WM (or a dry maze where they learn to find a hidden food reward) but are activated when rats are learning to navigate through space in two different contexts [51]. A similar function in contextual recognition was attributed to Neo-DGNs using genetic approaches and fear conditioning (see discussion [98]). Finally, DGNs generated during the adolescent period (Ado-DGNs, PND28) and not those born during the neonatal (PND7 or PND14) or embryonic (Embryonic day ED18.5) periods were found to be recruited by spatial learning in the WM but only within a critical time-window [52]. By optically silencing Neo-DGNs, Ado-DGNs and Adu-DGNs, we confirmed that spatial memory consolidation depends on Ado-DGNs (not Neo-DGNs) and on Adu-DGNs when animals become older [52]. We then focused on the process of reconsolidation of remote spatial memories and found that chemo-silencing Adu-DGNs (not Neo-DGNs) impairs the stabilization of the trace after reactivation [99]. As mentioned earlier, memories appear to be easily and rapidly forgotten in juveniles compared to adults. However, these memory are not lost as the opto-stimulation of tagged Juv-DGNs (PND17) ensembles leads to recovery of an engram when animals have reached adulthood [40]. However, this engram is qualitatively different (less cortical engagement) compared to the equivalent representation in adult animals, again suggesting that memory processing by Dev and Adult DGNs is different.

More indirectly, we have recently observed that suppression of *Rnd2*, a small rhoGtpase, in DGNs born in neonates (Neo-DGNs, PND1) has no effect on anxiety-like behavior whereas its deletion in Adu-DGNs exacerbates anxiety-like responses [100]. These facts indicate, first, that ablating DGNs neurons produces opposite deficits compared to those observed after adult hippocampal lesion [90, 101], and, second, that different cohorts of DGNs may play different and complementary roles in anxiety-like responses, as observed in social behavior and spatial learning. An alternative is suggested by a recent study showing that activation of embryonic neurons (Emb-DGNs) suppresses anxiety-like behaviors whereas their chronic inhibition or ablation increases anxiety-like behaviors [102]. These results are in the same line as those described for Adu-DGNs [81] but given that manipulation of Emb-DGNs regulates AHN [102], their specific role in anxiety remains to be disentangled. Interestingly, in line with our hypothesis, it has been shown that neurons born in the olfactory bulb during adolescence (PND42) control innate fear responses to predator odor, while adult-born ones (born at PND70) appear to control the acquisition of novel appetitive odors. Thus, newly added neurons may prime novel preferences without erasing critical F1 responses [103]. Altogether these data support the contention that DHN and AHN play complementary functions.

Now from a morphological point of view, beside their location in the GCL many differences have been described between the cell populations of the DGC at the level of their dendritic arbor. Thus Dev-DGNs were reported to mature faster than Adu-DGNs [104–107]. In mice, Emb-DGNs develop at least two primary dendrites and exhibit a short dendritic length with a wide branching angle while Neo-DGNs are distinguishable by their longer total dendritic length (with more nodes) and shorter trunk [107]. In contrast, the dendritic characteristics of Juv-DGNs (PND21) and Adu-DGNs were indistinguishable [107]. In rats, Neo-DGNs have more primary dendrites, a broader branching angle and more ramifications proximal to the soma compared to Ado-and Adu-DGNs; these 2 populations are quite similar except that the number of high-order dendrites is higher in Adu-DGNs compared to Ado-DGNs [52]. Adu-DGNs are also characterized by higher spine density, larger mossy fibers boutons (MFBs) and longer filopodia length compared to Neo-DGNs [108]. These distinct morphological features have most probably important functional implications in determining the signals each neuron receives and integrates, as well as the strength and directionality of the synaptic transmission [109–113]. In this vein, it has been shown that an early birthdate specifies DGN physiology and connectivity [113]. Traditionnaly, it is believed that Adu-DGNs exhibit a higher plasticity (lower threshold for long-term potentiation) only within a critical time window [114, 115]. However, recent evidences indicate that their plasticity extends over several months as indicated by enhanced responsiveness following in vivo LTP compared to Dev-DGNs [116]. In support of this long lasting plasticity of Adu-DGns, we have shown that Adu-DGNs (and not Dev-DGNs) exhibit the unique property to reshape in response to learning [117, 118], further reinforcing the contention that Adu-DGNs are distinct from Dev-DGNs and that they may provide a unique source of plasticity to elaborate adaptive behavior. Interestingly, a recent study has elegantly estimated that although the rate of neuronal production decreases with age, ~50% of total number of DGNs and of dendrites are added in adulthood [108]. Altogether, given that Adu-DGNs seem to be more plastic then Dev-DGNs, their implication in adult behavior may surpass the implication of Dev-DGNs.

In summary, we propose according to the Baldwin effect hypothesis that F1 capacities are installed and maintained by DHN neurons in a repertoire of basic and rigid stimuli-to-behaviors setups whereas F2 capacities, which require to establish (or “map”) the relationships between infinitely varied stimuli––both in space and time––, qualified emotions, and flexible behaviors, are sustained by AHN. What could be the mechanisms by which AHN may bind together multiple types of information? We hypothesize that Adu-DGNs may exert this function by maintaining total synaptic weights constant through concurrent synaptic potentiation and depression. Supporting this view, Adu-DGNs have been shown to control the activity of the DG and related structures (CA3/CA1, frontal cortex) [61, 105, 119–121] along with long term potentiation and depression [122, 123].

## Is adult hippocampal neurogenesis involved in the pathophysiology of mental disorders?

Both DHN and AHN constitute key elements in building a behavioral response adapted to the environmental conditions––DHN through the early establishment of a repertoire of basic elements or modules necessary for this adapted response and AHN through the lifelong binding of these modules in appropriate combinations. When impaired, these two functions may be involved in mental disorders. An important distinction is to be made between impaired F1 and impaired F2, respectively corresponding to DHN and AHN.

Following our Baldwin interpretation of HN, F1 is “hardwired”; it corresponds to a developmental, phylogenetic program for a repertoire of behaviors. F1 consists in a series of traits that strongly (but not exclusively) depend on genetics and to a lesser extent on environmental factors. When impaired, it is likely to lay the scene of developmental disorders and to induce developmental vulnerability that conditions the occurrence of mental disorders later in life. By opposition, when F1 is optimal and DHN levels fit the environmental constraints, it is expected that individual have developmental resilience.

As opposed to F1, F2 is “software-like” and corresponds to how the user of a given organism in a given environment makes the best of its experience of past life events. It is a series of traits that strongly depends on environmental factors and to a lesser extent on genetics. Typically, it depends on genes that control the level of AHN or the integration of new neurons in the brain [124]. But it much more depends on how the user conducts its experience and on the way he/she binds life elements together. When impaired, F2 triggers mental disorders such as anxiety disorders, addiction, PTSD, and possibly major depression (MDD). The variable susceptibility of individuals to impairment of F2 defines a continuum from adult resilience [125] to adult vulnerability and we hypothesize that it should not rely on the same biological substrate as developmental vulnerability.

**Fig. 2:**
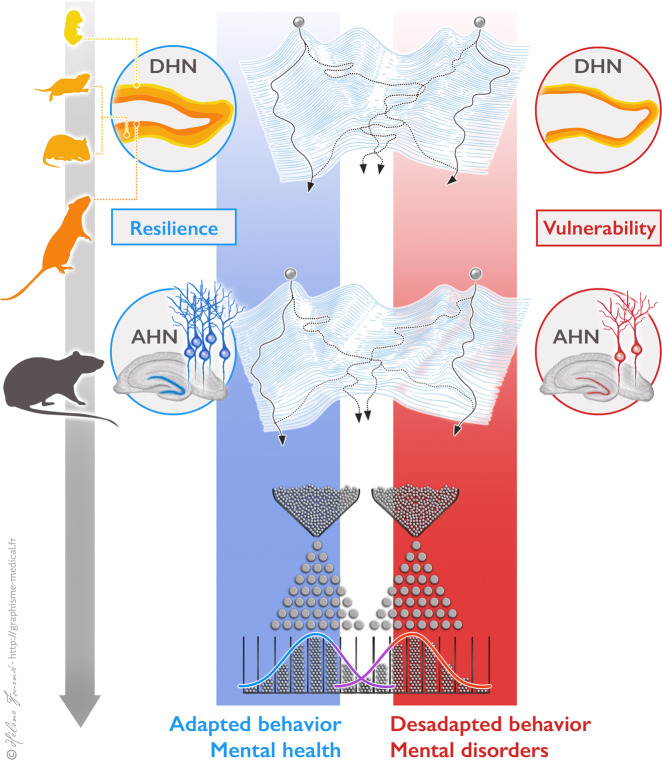
Developmental trajectories leading to mental health or mental disorders. Trajectories of individuals’ adapted (mental health) or maladapted (mental disorder) behavior follow three stages. The first one relies on DHN, and the second one on AHN. Both depend on genetics (that determine the shape of a Waddingtonian landscape) and environmental factors (determining the initial position of the ball and the direction it actually takes on forking paths). However, they do not depend on the same genes or events. They respectively end down in developmental or adult resilience/vulnerability to maladaptation. The probability to be oriented toward a given phenotype is indicated by the lines format (high probability: solid lines; low probability: dashed lines). The third stage gathers exposure to a series of mostly random triggering events of mental disorders, represented as nails in a Galtonian quincunx. Depending on both its previous trajectory and its reactions to these obstacles, the ball is more likely to fall on the left-hand or on the right-hand of the quincunx, following a Gaussian distribution law.

Our proposal thus distinguishes levels of developmental vulnerability/resilience that are programmed during development, and levels of adult vulnerability/resilience, which result from the buildup of life experience. All combinations of vulnerability and resilience are theoretically possible (Fig. 2) and this quadripartite distinction, which replaces the usual vulnerability/resilience distinction, is a direct consequence of the Baldwin interpretation of AHN. In short, the level of adaptability of an animal to a changing environment both depends on its repertoire of F1 abilities, dictated by DHN levels, and on its F2 ability to use them adequately, which relies on AHN levels.

In the rest of this section, we will explore how this distinction fits with experimental data and what novel experimentations it suggests.

### Experimental evidence necessary to link AHN to mental disorders

Previous data showing that ablating AHN leads to behavioral disturbances often associated with mental disorders has paved the way to propose that AHN could be involved in the pathophysiology of these disorders. An important amount of data has accumulated on this topic and has been the subject of excellent reviews [15, 17, 18, 79, 126–146]. However, we argue here that because ablation is usually performed on a healthy brain and sometimes with aspecific methods, it presents 3 main drawbacks:

First, it is not always clear whether behavioral deficits depend on impairments of DHN or AHN. When manipulations of neurogenesis are performed during development and deficits appear before adulthood, these can of course be attributed to an impairment of F1/DHN (some aspects of developmental mental disorders). However, when manipulations are done during development and deficits are analyzed in adulthood, it is not clear whether these are a consequence of ablating DHN or a secondary consequence of impairing AHN, through alterations of the neurogenic niche for example, and so far most manipulations targeting development also impact AHN. For example, neonatal irradiation of the dorsal hippocampus produces hyperactivity and facilitates active avoidance learning [147] which is similar to what is seen in animals hippocampectomized in adulthood;[148] This is consistent with the observation that behavioral inhibition emerges early during development and does not rely on AHN. Another interesting example comes from neonatal lesions of the ventral hippocampus. Indeed, this procedure leads to profound behavioral disturbances: sensorimotor gating and latent inhibition deficits, decreased social interactions associated with aggressive behavior, impaired social recognition memory, diminished sensitivity to rewarding stimuli, hyper-responsivity to drugs and environment (novelty-induced hyperactivity, hyper-responsivity to stress), enhanced acquisition of sucrose and cocaine self-administration [149–151]. Interestingly, among these deficits, the negative-like symptoms emerge before puberty indicating that they are linked to an alteration of F1 and embryonic and/or neonatal hippocampal neurogenesis. The positive-like symptoms on the other hand appear post-puberty and may result from F1 and adolescent hippocampal neurogenesis or from F2 and AHN. These examples highlight that much work is still needed to better attribute a specific role for DHN in the ontogeny of behavior and/or the appearance of psychopathology, and approaches such as specific tagging of DGN at a given period of development, using pharmaco-or opto-genetics, and later silencing or activating tagged cells without changing the activity of the whole network should be developed.

Second, in a lot of cases the consequences of ablating AHN do not model the full extent of a disease. Indeed, behavioral changes may result from a combination of altered neurogenesis and adverse experience and may not be visible after ablating AHN under normal conditions. In accordance with this idea, mice lacking AHN show normal behavior when naïve, while they exhibit increased depressive-like behavior when exposed to stressful events [123].

Third, although ablation studies provide evidence for a link between AHN and mental disorders, they do not allow determining whether changes in AHN are adaptive responses to the different pathophysiological conditions, are part of the pathophysiology that contributes to the disease, or both. From a conceptual point of view, showing that altered AHN recapitulates the symptoms of a disease indicates that disturbances of AHN may be a sufficient condition, not a necessary condition, to explain behaviors assimilated to mental disorders; such demonstration also requires showing that manipulation of AHN in a diseased brain alleviates the disease-associated behavioral deficits. Although some attempts have been made to provide such demonstration, the main issue raised by this approach is that of the animal models.

### The animal model issue

In the investigation of mental disorders, animal models are both indispensable and problematic. The match or mismatch has traditionally been described in terms of face, construct and predictive validity [152]. Contrarily to a widely shared opinion, the problem is not that “mental” disorders cannot be modeled in animals. In the light of the Baldwin hypothesis, all animals that must accumulate an experience of their environment to be adapted to it are likely to be subjected to the risk of an equivalent of human ‘mental disorders’. The problem is rather with their neurobiological “locus of control”, that is, according to two philosophers’ definition [153], the place where a particular function sits, where a problem occurs when the function goes awry, and where it can be intervened upon; indeed, this place is still vaguely localized and hardly decomposed [153] in the case of mental disorders as compared to many other situations in medicine, which triggers ambiguous use of terms [154, 155]. In contrast, the question with the investigation of AHN is whether forms of dysfunction in a known locus of control are associated with the equivalent of mental disorders in animal models. In the precise terms proposed by Belzung & Lemoine [156], mechanistic validity, that is the resemblance of mechanisms in model and target, is reasonably good when investigating AHN and its disturbance. However, the Baldwin hypothesis predicts that important parameters will be associated with species-specific level of demand on ontogenetic adaptation, and thus are likely to reduce “homological” and “pathogenic” validity, i.e., the cross-species similarities between initial states and extrinsic events, respectively, that lead on the path of maladaptation. Indeed, in a broader and modular approach to the underpinnings of mental disorders that does not rely on disorder-based categories, such as the RDoC (Research Domain Criteria) advocates [157], there are strong reasons to think that the AHN “module” is a component involved in many mental disorders, but no strong reason to specifically associate some forms of dysfunction to fine-grained distinctions between categories of human pathological manifestations. That said, it is thus not surprising that altered AHN is associated with different mental disorders in animal models, especially those linked to altered emotional states such as addiction, depression or PTSD, and the following parts will review the existing literature on their association. It should be noted that although levels of AHN have been linked to variations in anxiety-like behavior, to the best of our knowledge, there is no animal models to date reproducing specific symptoms of the anxiety disorders listed in the *DSM-5*, i.e., separation anxiety disorder, selective mutism, specific phobia, social phobia, panic disorder, agoraphobia, and generalized anxiety disorder. As a consequence, the putative involvement of AHN in the pathophysiology of these disorders can at best be extrapolated from studies manipulating AHN but elucidating its causal contribution will depend on the development of adequate and relevant models.

#### AHN in models of drug addiction

Drug addiction has been considered as an aberrant form of learning mediated by maladaptive recruitment of the hippocampus that is important in the formation of drug–context associations and in the mediation of drug-taking and drug-seeking behaviors. So far the role of AHN has been studied in both drug taking and drug seeking using a self-administration (SA) paradigm. Classically, animals have to learn to administer drug infusions by providing a response in a specific device (either a hole into which the animal has to insert its nose or a level that it needs to press). It has first been shown that suppression of AHN by irradiation before drug exposure increases cocaine SA delivered either as fixed doses or escalating work-effort contingencies. In addition, suppression of AHN after animals acquired cocaine SA behavior renders them more resistant to extinguishing drug-seeking in the absence of drug [92]. Using a transgenic approach to deplete AHN, we found that while acquisition of cocaine SA was not impaired motivation for the drug was increased: AHN depleted mice worked harder to obtain the drug compared to control mice. In addition, a higher SA reinstatement was induced by the presentation of a cocaine-associated cue [16]. AHN depletion also increased the propensity to self-administer other drugs such as morphine in irradiated rats [86]. As suggested earlier for drug conditioning, we propose that removing Adu-DGNs leads to an excessive binding (between the cues and the drug) leading to an inability to recall properly the context in a novel condition (no drug delivery).

Having said that, modeling such a multifaceted and multi-step disease is difficult. Indeed, addiction is not just taking drug; it is a non-adaptive drug use and all drug users do not face the same individual risk of developing addiction. But given that addiction develops after protracted periods of controlled drug use, models should allow the study of the long-term shift from controlled drug use (the so-called recreative use) to addiction (loss of control) [158]. So far this aspect has not been examined. Furthermore, whether Adu-DGNs and Dev-DGNs play a similar or distinct role in addiction is still a matter of debate. Although the specific role of Dev-DGNs has not been investigated, temporary inactivation of the hippocampus was found to block context-induced, cue-induced, and cocaine-induced reinstatement of cocaine-seeking [159, 160], and specific inactivation of entorhinal cortex to DG projections was reported to decrease context-induced reinstatement of heroin seeking [161]. Because these results are opposite to those described after specific AHN ablation, they support the contention of a differential role of DHN and AHN in addiction.

#### AHN in models of depression

The Baldwin hypothesis complements the common diathesis model, according to which vulnerability is built up during development and triggered by stressful life events. Indeed, it suggests that developmental and adult vulnerability to depression may depend on different genes and on different stressful events. From a neurobiological perspective, the current view is that alteration of AHN is not sufficient to induce depressive-like behavior in models of major depression [81, 162], but that it is necessary to some of the therapeutic effects of antidepressants (ADs) [163]. However, studies are not consistent, and protocols and endpoints differ. The Baldwin hypothesis suggests that ablation of AHN should indeed not have an etiologic effect when circumstances do not require the building up of an adapted response, but should when they do. Additionally, it predicts that if ADs indeed have an effect on neurogenesis, they can help restore some conditions of normal adaptive function, namely the ability to bind memory of life events, emotions and behaviors, but not other conditions, for instance, the right ways to bind them in order to provide an adapted response (adult resilience). The Baldwin hypothesis likewise predicts that ablation of DHN should induce a permanent disposition to depressive-like behavior (developmental vulnerability) and thereby play an etiologic role in adult depression, and that if ADs have an effect on DHN, they can restore some conditions of normal adaptive behavior in adults. Most of these predictions remain to be tested: in particular, it would be crucial to observe possible behavioral differences in adults between DHN-based and AHN-based vulnerability. Supporting this hypothesis, a recent study revealed that the effects of ADs depend upon neurons formed during development [164]. Indeed, deleting Serotonin 1 A receptors (5HT1AR; a receptor required for fluoxetine response) specifically from DGNs during development abolished the effects of fluoxetine on behavior (Novelty Suppressed Feeding, Elevated plus maze, Forced swim test). By contrast, deleting 5HT1ARs only in Adu-DGNs did not influence fluoxetine responses. These results indicate that 5HT1ARs on Dev-DGNs are necessary and sufficient to mediate the effects of fluoxetine on some behavior and suggest that DHN (F1) may be involved in the etiology of depressive-like behavior. However, the acute manipulation of Adu-DGNs’ activity using chemogenetics has also been shown to regulate depression-like responses [83], opening new avenues on the role of AHN in the aetiology of this disease.

#### AHN in models of PTSD

PTSD has been until recently considered as an anxietydisorder driven by exposure to traumatic, i.e., highly stressful, frightening or distressing, events. As a consequence the different animal models that were developed focused exclusively on the persistence of a strong fear memory in response to a traumatic event. In line with this framework, it has been proposed that AHN is involved in PTSD in virtue of its “putative” role in pattern separation [15]. This property of newborn cells to discriminate similar contexts would prevent generalization of fear in healthy individuals exposed to traumatic events. However, as said before, fear is a normal emotional state serving adaptive function and fear learning per se cannot be considered as a pathological fear memory. In addition, PTSD is a maladaptive fear memory which is characterized by a relative amnesia for the context in which the traumatic event took place associated with intrusive recollection of the trauma in a safe environment [165]. So considering that “generalization” is a core symptom is a shortcut and does not capture PTSD complexities, and very few models address the decontextualisation of memory. In addition, as discussed earlier [165], in most studies stressed animals are compared to control non-stressed animals, which does not allow determining whether the changes observed are linked to normal adaptive responses to stress exposure or to PTSD. Indeed, it is well know that not all individuals exposed to a trauma develop a PTSD [166]. This individual variability may be linked to a defect in NG. The Baldwin hypothesis suggests that stress during development may orient toward a developmentally vulnerable phenotype and/or that a low level of AHN may trigger PTSD in vulnerable animals exposed to trauma. The recent development of more relevant models that capture the core hippocampal-linked symptoms of PTSD-like memory deficits [167] and that allow predicting adult vulnerability and resilience [168] will certainly help testing this hypothesis.

Altogether, and keeping in mind that the currently available animal models are not flawless, these examples clearly indicate that although AHN has been linked to some features of mental disorders, its specificity, and thus the exclusion of a role for DHN, has not been clearly demonstrated. In line with the RDoC initiative, a broader analysis of the previous pathologies indicates that they are highly comorbid conditions that are characterized from a psychological standpoint by an altered reactivity to emotional stimuli. This shared process suggests common pathophysiological mechanisms, which could provide a better framework for linking AHN with mental disorders. Notwithstanding the specifically human sense in which ‘stress’ is now understood in the DSM5, all these pathologies have been linked to one common triggering event: stress, in the classic, neurobiological meaning of the term. Indeed, major depressive disorder, bipolar disorder, anxiety and panic disorders, PTSD and addiction can all be classified, in neurobiology, as stress disorders where key neural circuits that regulate stress reactivity are not functioning optimally. This dysregulation might include enhanced reactivity to threatening stimuli, decreased ability to terminate the stress response, and/or suboptimal coupling between internal affective states and external environment [169]. Together with the role of stress in modulating AHN [170], this observation indicates that a more consistent and better approach could be to analyze the involvement of AHN in stress-induced behavioral pathologies.

### Involvement of AHN in stress-induced disorders

Using various models and paradigms to mimic chronic stress exposure in humans, it was almost consistently reported that the intensity of the behavioral and endocrine response to stressful events is related to the levels of AHN. For instance, lowering the activity of Adu-DGNs by chemogenetics increases susceptibility to the anxiogenic effects of a subthreshold regimen of social defeat that does not induce any behavioral alterations in control mice [171]. Conversely, genetic enhancement of AHN by blocking the naturally occurring death of half of the newlyborn cells (iBax mice model) before chronic corticosterone (CORT) administration or chronic social defeat exposure appears sufficient to prevent the development of behavioral disturbances such as increased anxiety-like and depression-like responses [171, 172], indicating that high levels of AHN promotes resilience to the negative effects of chronic stress. Interestingly, using the same experimental tool to enhance AHN once the stress regimen had already engendered physical deficits is sufficient to reverse the effects of UCMS on some behavioral responses, and to normalize the HPA axis activity [173], indicating that high levels of AHN not only prevent but can also rescue behavioral deficits induced by chronic stress exposure.

This relationship between AHN and stress-induced disorders is further emphasized by studies showing that the beneficial effects of treatments on stress-induced depressive symptoms require neurogenesis. Thus while enriched environment (EE) can rescue the submissive and depression-like behaviors adopted in response to chronic psychosocial stress in control mice, it is inefficient in mice genetically deficient for AHN [174]. On the same line, irradiation prevents behavioral improvement consecutive to monoaminergic AD treatment in chronically-stressed mice (UCMS paradigm or chronic CORT treatment) [175, 176].

From a mechanistic perspective, it was suggested that Adu-DGNs promote resilience to the anxiogenic effects of chronic stress by exerting an inhibitory control on a population of stress-responsive DG cells [171], and that decreasing AHN alters the negative feedback control of glucocorticoid release and promotes escape from the dexamethasone suppression test, indicating that optimal levels of AHN are necessary for an adequate endocrine response to stress [123, 177, 178]. Interestingly, it should be reminded that AHN is itself strongly regulated by stress and glucocorticoids forming a loop whereby stress, by inhibiting AHN, could lead to enhanced stress responsiveness and altered behavior.

Taken together, these data make a strong case to propose that pre-existing optimal levels of AHN could protect from developing mental disorders by buffering the negative consequences of stress as a precipitating factor of mental disorders. Although this hypothesis has been raised and commented several years ago [179], it still awaits further experimental testing. These data also raise a couple of questions: (i) If optimal levels are required to buffer the negative consequences of stress exposure, how were these optimal levels acquired, or in other words why are levels non optimal in some individuals? (ii) if AHN involvement requires inhibition of stress-responsive mature DGN [171], what is the respective contribution of DHN vs AHN in the behavioral deficits induced by stress?

### Importance of the developmental niche in determining AHN levels and emergence of psychopathology

The brain of altricial species (humans, rodents) is characterized by a protracted development with considerable amounts of neurogenesis, synaptogenesis, and gliogenesis extending from fetal life to several years after birth; furthermore, cellular events that fine-tune the brain, like cell death, dendritic pruning, and synapse elimination, dominate during postnatal life and altogether, these extended structural changes provide opportunities for life experiences to sculpt brain development. Thus, the ‘fetal programming hypothesis’ [180], the ‘developmental programming hypothesis’, and the ‘Developmental Origins of Health and Disease (DOHaD) hypothesis’ [181] specifically state that, during these critical or sensitive periods of development, a disturbance in environmental factors has organizational effects on biological systems (central and autonomic nervous system, neuroendocrine, cardiovascular, and immune systems) in order to react and adapt to environmental influences. The resulting changes will enhance susceptibility to somatic diseases and mental health problems which, in interaction with genetic liabilities, will determine ultimate health status.

In accordance with these hypothesis, hundreds of human studies ranging from epidemiological studies of famine or war to prospective cross-sectional and case-control analyses have isolated early life adversity as a prominent risk factor for mental disorders in adult humans [182, 183]. For instance, according to the Adverse Childhood Experiences (ACEs) studies, exposure to one or more maltreatment-related ACEs accounts for 54% of the population attributable risk (PAR) for depression, 67% of the PAR for suicide attempts and 64% of the PAR for addiction to illicit drugs. Exposure to five or more ACEs was further associated with a 2-, 3-, 10- or 17-fold increase in risk for receiving prescription of an anxiolytic, antidepressant, antipsychotic or mood-stabilizing medication, respectively [184].

In light of the previous experimental evidence linking AHN with these mental disorders, many studies have addressed the programming effects of early life adversity on AHN levels [170, 185], and have consistently reported that stress applied in utero (prenatal stress; PreS) or during the early postnatal period (early life stress; ELS) decreases AHN levels in rodents and primates throughout life (see for example [186–196]). Although in some instances the effects of PreS or ELS are not visible under baseline conditions, interferences with AHN process are visible in response to challenging conditions, as for example ELS prevents the stimulatory effects of exercise on AHN [197], and alters AHN’s sensitivity to stress [198] or to AD treatment [199]. Taken together, these data thus strongly implicate AHN in the pathophysiology of mental disorders consecutive to early life adversity. However, because early life events interfere with development, they can alter the neurogenic niche as early as the embryonic stage [200, 201] thereby impairing DHN [202, 203] as well. As a consequence AHN is not specifically targeted and altered production of Adu-DGNs could be only a secondary consequence of alterations in the primitive neurogenic matrix. Supporting this limitation, it was shown that if early life stress can rewire the DG throughout the life of the individuals, its long-term consequences can be prevented by positive manipulations occurring during development. For example fluoxetine treatment for the first 3 weeks of life normalizes DHN levels in adolescent mice previously exposed to PreS [204], and neonatal handling prevents PreS-induced decrease in AHN [205] and memory function [206] most probably through normalization of the HPA axis activity [207, 208]. On the same line, physical activity during adolescence can prevent the occurrence of spatial memory deficits in rats previously exposed to PreS [209] and EE starting in adolescence prevents the enhanced fear and decreased AHN observed in animals exposed to pre-pubertal stress [210].

Altogether these studies indicate that along with alterations of DHN, manipulation of the neurogenic niche may be involved in the long-term effects of early life events, and consequently, the relative contribution of DHN and AHN to pathology remains unclear. One way to sort out their involvement is to analyze the emergence of behavioral disturbances across development and to evaluate the consequences of normalizing AHN in animals previously exposed to early life events: if behavioral disturbances are alleviated, this implicates AHN in the early life stress-induced behavioral defects; if not, it suggests DHN is determinant in the long-term behavioral consequences. Unfortunately we couldn’t find in the literature studies specifically targeting AHN in animals previously exposed to PreS or ELS, which highlights the need for further studies.

In summary, although evidence has accumulated to incriminate AHN in the pathophysiology of mental disorders, the lack of studies specifically addressing its contribution respective to that of DHN in mental disorders elicited after adverse early life events leaves open the question of its specificity. In accordance with our general hypothesis, and awaiting experimental testing, the available data lead us to propose that adverse early life environment, along with genetic predisposition factors, may define an initial fragile state characterized by low DHN and low AHN levels. Later exposure to negative stressful influences (second hit) that AHN cannot buffer may then trigger the emergence of a pathological state (Fig. 2). In this framework, even though the mechanisms are not yet elucidated, developmental exposure to AHN-enhancing events that could prevent or reverse the negative effects of adverse life events, may, by restoring normal levels of AHN, determine an adult resilient state. Supporting this hypothesis, exposure to beneficial stimulations in early life was found to counteract the negative consequences of PreS on AHN in animal models [191, 204, 205, 211, 212] and to alleviate some of the negative consequences of early stress in humans [213].

## Conclusion

Animals can be genetically hardwired to automatically respond to a limited set of stereotyped forms of challenge, but genetic variations cannot possibly be selected to respond to all environmental challenges they have to face during their life. The reason is that most of these challenges stem from a broad variety of life events which requires fine-grained adaptation, relying on memory, association and dissociation––in short, what can be called “binding”––and is responsible for preparatory tasks for inferential reasoning. Natural selection has equipped many animals with an apparatus of capacities to face the unpredictable variety of situations they will encounter during their life. Baldwin called this a situation of “ontogenetic adaptation”. According to him, it is based in part on non-genetic transmission through breeding or education and has a transgenerational dimension [214]. It is likely that some traits of such non-genetic individualization do not depend on individual life trajectories so much as on how ancestors learned, and taught, to behave, a process that is likely to take place even in rodents [215, 216]. But mainly, we can consider this ontogenetic adaptation to be based on what befall an individual and how it is interpreted. In short, capacities to adapt are hereditary; some rules are transmitted through learning; but the adjustment of behaviors is not and cannot be genetic. This “Baldwin effect” is in turn responsible for pressure put on individuals to adapt rather than on the species to evolve. This pressure put on individuals explains individual variability beyond genetic variability.

The evidence is strong that these adaptive capacities in general are supported by neurogenesis in the DG. Most authors that have discussed the specific function of AHN have insisted on aspects of the Baldwin hypothesis, like flexibility, individualization, the role of an enriched niche [11], or on the so-called double neurogenic niche hypothesis [217]. The Baldwin hypothesis proposes a unifying framework for all these important contributions. In particular, it suggests that the capacities that underlie ontogenetic adaptation (sensus Baldwin) consist in two sets:

- F1 capacities that are acquired first during development and are more strongly determined by a genetic program. They provide a first layer of general adaptive behaviors.
- F2 capacities that are exerted throughout adult life and depend more on life events and on how individuals have faced them. They provide a second layer of more specific adaptive behaviors.

This in turn strongly suggests that a sharp distinction should be made between the respective roles of DHN and AHN in the onset of adapted or maladapted behaviors. Conceptually, we proposed to distinguish between (DHN-related) developmental resilience or vulnerability, and (AHN-related) adult resilience or vulnerability. They should correspond to different behavioral or cognitive/emotional abilities. Developmental resilience is expected to be associated with a repertoire of basic reactions––e.g., how intensely and how easily an animal may experience fear––and to remain mostly invariable throughout life. Adult resilience is expected to be associated with how adults bind memories, emotions and behaviors depending on past and present experience––e.g., determining when fear should or should not be experienced––and be susceptible to some degree of change during life. Although there already is solid evidence that AHN and DHN and their impairments play different roles in the risk of adapted or maladapted behaviors, the Baldwin hypothesis we propose suggests that it should be explored further. In particular, not many experiments have compared the respective effects of the ablation, enhancement or limitation of hippocampal neurogenesis during development and in adulthood on the onset of behaviors comparable to human mental disorders.

It is probably too early to state what the consequences of the theoretical framework proposed in this review are for clinical and therapeutic development. Suffice it to say here that it potentially questions the relevance of some distinctions between mental disorders, and suggests hypotheses about their underlying mechanisms and what can be done to relieve them. The Baldwin hypothesis also states that mental disorders are a paradoxical, but necessary adverse effect of having the kind of flexible minds humans have. Humans build complex rules that bind behaviors together in social life, in order to elaborate a sophisticated collective adaptive response that improve their chances of survival in the face of unpredictable events. At the same time, these rules are hard to learn and it falls on individuals to acquire them. Events like catastrophes and pandemics demonstrate how human capacities of adaptation save lives and at the same time generate stress. So, just as some of us can make the best out of that and wonderfully adapt to many circumstances, it comes for others with a burden of developmental and adult vulnerability.
